# Vitamin D status in ANCA-associated vasculitis

**DOI:** 10.1093/rap/rkad021

**Published:** 2023-02-10

**Authors:** Irena Doubelt, David Cuthbertson, Simon Carette, Nader A Khalidi, Curry L Koening, Carol Langford, Carol A McAlear, Larry W Moreland, Paul Monach, Philip Seo, Ulrich Specks, Kenneth J Warrington, Peter A Merkel, Christian Pagnoux

**Affiliations:** Vasculitis Clinic, Mount Sinai Hospital, Department of Rheumatology, University of Toronto, Toronto, ON, Canada; Health Informatics Institute, University of South Florida, Tampa, FL, USA; Vasculitis Clinic, Mount Sinai Hospital, Department of Rheumatology, University of Toronto, Toronto, ON, Canada; Division of Rheumatology, McMaster University and St. Joseph’s Healthcare, Hamilton, ON, Canada; Division of Rheumatology, University of Utah Hospital, Salt Lake City, UT, USA; Division of Rheumatology, Cleveland Clinic, Cleveland, OH, USA; Division of Rheumatology, Department of Medicine, University of Pennsylvania, Philadelphia, PA, USA; Division of Rheumatology, University of Pittsburgh, Pittsburgh, PA, USA; Division of Rheumatology, VA Boston Healthcare System, Boston, MA, USA; Division of Rheumatology, Johns Hopkins University, Baltimore, MD, USA; Division of Pulmonary and Critical Care Medicine, Mayo Clinic College of Medicine and Science, Rochester, MN, USA; Division of Rheumatology, Mayo Clinic College of Medicine and Science, Rochester, MN, USA; Division of Rheumatology, Department of Medicine, University of Pennsylvania, Philadelphia, PA, USA; Vasculitis Clinic, Mount Sinai Hospital, Department of Rheumatology, University of Toronto, Toronto, ON, Canada

**Keywords:** vitamin D, ANCA-associated vasculitis, cohort study, granulomatosis with polyangiitis, eosinophilic granulomatosis with polyangiitis, microscopic polyangiitis

## Abstract

**Objective:**

Vitamin D might participate in the pathogenesis of several immune-mediated diseases, but few related data are available for ANCA-associated vasculitis (AAV). In this study, we analysed the association between vitamin D status and disease in patients with AAV.

**Methods:**

Serum levels of 25(OH)D_2/__3_ were measured in 125 randomly selected patients with AAV [granulomatosis with polyangiitis (*n* = 50), eosinophilic granulomatosis with polyangiitis (*n* = 50) or microscopic polyangiitis (*n* = 25)] enrolled in the Vasculitis Clinical Research Consortium Longitudinal Studies at the time of enrolment and a subsequent relapse visit. Sufficient, insufficient and deficient vitamin D status were defined as 25(OH)D_3_ levels >30, 20–30 and ˂20 ng/ml, respectively.

**Results:**

Seventy of 125 patients (56%) were female, with a mean age of 51.5 (16) years at diagnosis; 84 (67%) were ANCA positive. Mean 25(OH)D was 37.6 (16) ng/ml, with vitamin D deficiency in 13 (10.4%) and insufficiency in 26 (20.8%). In univariate analysis, lower vitamin D status was associated with male sex (*P* = 0.027) and disease activity (*P* = 0.047). In univariate and multivariate analyses, deficient vitamin D status was associated with disease activity (*P* = 0.015). Mean 25(OH)D status in the 21 patients with a subsequent relapse did not differ between baseline and relapse visit [37.8 (16) *vs* 38.0 (10) ng/ml, respectively; *P* = 0.92].

**Conclusion:**

Most patients with AAV have sufficient 25(OH)D levels, although those with lower vitamin D status were more likely to be male and to have active disease. Whether optimization of vitamin D status alters disease manifestations or activity in AAV remains to be determined.

**Trial Registration:**

Vasculitis Clinical Research Consortium (VCRC) Longitudinal Study (LS), NCT00315380, https://clinicaltrials.gov/ct2/show/NCT00315380

Key messages

- Over one-third of patients with ANCA-associated vasculitis in this cohort had vitamin D insufficiency/deficiency.
- Lower 25(OH)D levels were associated with male sex and disease activity at the cohort level.
- The potential impact of vitamin D supplementation on ANCA-associated vasculitis disease activity remains uncertain.

## Introduction

Although the role of vitamin D in bone health is well established [1], knowledge of its potential extra-skeletal effects continues to grow and is controversial. Vitamin D has been shown to exert some influence on the cardiovascular, respiratory, nervous and endocrine systems [1] and is also thought to be involved in immunomodulation in both innate and adaptive immune responses [2]. Furthermore, vitamin D deficiency [low concentration of serum 25(OH)D_3_)] is suspected to participate in the pathogenesis of several autoimmune conditions and to be a marker of inflammation or outcomes in some diseases [2].

In obesity and IBD, an inverse relationship with 25(OH)D_3_ levels and markers of inflammation was reported [2]. Insufficient and/or deficient vitamin D status is also commonly found in inflammatory arthritis [3, 4] and some CTDs [5, 6]. However, controversy remains in interpreting these findings, particularly in defining optimal vitamin D status, and determining whether inflammation reduces vitamin D levels or whether improving vitamin D status reduces inflammation.

Data on vitamin D status in vasculitis are scarce, with only a few small-sample studies in Behçet’s disease [7], large-vessel vasculitis [8] and various medium- to small-vessel vasculitides [9]. ANCA-associated vessel vasculitis (AAV) is a group of rare, heterogeneous, multisystem diseases causing inflammation of small blood vessels [10]. A single study in patients with AAV from Turkey found that vitamin D status was negatively correlated with BVAS, CRP and white blood cell count (WBC) [11], suggesting that vitamin D status might provide clinical utility for the management of AAV [11].

The aim of the present study was to determine the vitamin D status of patients with AAV and assess the correlation of this status with certain disease manifestations, disease activity and/or relapse.

## Methods

### Patients

The Vasculitis Clinical Research Consortium (VCRC) Longitudinal Studies are cohort studies that prospectively collect longitudinal clinical data and link biological samples in patients with various vasculitides across eight US and two Canadian sites. For the present study, data and sera from a random sample of 125 patients from the VCRC were analysed: 50 patients with granulomatosis with polyangiitis (GPA), 50 patients with eosinophilic granulomatosis with polyangiitis (EGPA) and 25 patients with microscopic polyangiitis (MPA).

Patients were ≥18 years old, with GPA, MPA or EGPA according to the ACR 1990 criteria [12] and/or the revised 2012 Chapel Hill nomenclature [10]. Patients were excluded if they had a known history of hypercalcaemia, primary hyperparathyroidism, sarcoidosis, hypervitaminosis D, Williams syndrome, other chronic inflammatory or infectious conditions, malabsorptive disorders, cancer, type 1 diabetes, liver disease or current pregnancy.

### Studied parameters

Demographics, main clinical manifestations of AAV (at baseline, i.e. enrolment in the cohort, and at a subsequent relapse visit if applicable), ANCA status, disease activity (remission *vs* active disease), laboratory measures (haemoglobin, WBC, platelets and CRP), history of relapse (before and after enrolment) and types of treatments received were extracted from the VCRC database. For sub-analyses, enrolment centres were grouped into two categories based on location: north (Toronto, ON, Canada; Hamilton, ON, Canada; Rochester, MN, USA; and Boston, MA, USA) and south (Philadelphia, PA, USA; Baltimore, MD, USA; Cleveland, OH, USA; Salt Lake City, UT, USA; and Pittsburg, PA, USA). The 25(OH)D level was also grouped into summer (June–October) and winter (November–May) seasons based on collection date.

Serum samples were obtained from the initial study visit for all study patients, and at the first subsequent visit with 2 years of enrolment during a relapse for ≤25 patients. The vitamin D levels, measured as the two most physiologically derived isoforms of 25(OH)D_3_ (animal derived) and 25(OH)D_2_ (plant derived), were analysed in duplicate by ELISA (Euroimmun 25-OH Vitamin D; PerkinElmer, Germany). Vitamin D status was defined as sufficient, insufficient or deficient, based on serum levels >30 ng/ml (˃75 nmol/l), 20–30 ng/ml (50–75 nmol/l) or <20 ng/ml (˂50 nmol/l), respectively [13].

### Study ethics

The VCRC cohort studies were approved by the Hospital Research Ethics Board committees at each participating VCRC site. All subjects in the VCRC provided written, informed consent for their participation before enrolment. The present study was approved by the Sinai Hospital ethic board (MSH REB number: 19-0039-E). The research was performed in accordance with the ethical principles of the Declaration of Helsinki.

### Statistical analyses

Quantitative variables are reported as the mean (s.d.) and compared using Student’s paired *t*-tests. Categorical variables are reported as count (percentage) and compared using χ^2^ tests or Fisher’s exact test, as appropriate. Predetermined variables analysed with respect to vitamin D status included: age, race, sex, geographical location, disease, ANCA status, haemoglobin, WBC, platelets, CRP, glucocorticoid use, kidney involvement, disease status (remission *vs* active) and history of relapse. Multivariate linear logistic regression analysis was used to look for association between vitamin D level and parameters identified as possibly linked with vitamin D status in univariable analysis (with a *P*-value ≤0.2). Statistically significant differences were defined as *P*-values <0.05. Statistical analyses were performed using Stata Software, v.12 (StataCorp).

## Results

### Demographic and clinical characteristics at enrolment

Characteristics of 125 patients at enrolment visit in the VCRC cohort are presented in Table 1. The majority of the cohort was female (56%), white (92.8%), with an average age at diagnosis of 51.5 (16) years [age at enrolment of 55.4 (15) years]; and 83 (66.4%) patients were from northern sites. ANCA status was recorded as positive (ever) in 84 (67%). Lung (80%), constitutional (74%), musculoskeletal (65%), renal (46%) and ENT involvement (42%) were the most common clinical manifestations.

**Table 1.** Clinical manifestations at study enrolment for 125 patients with ANCA-associated vasculitis

Characteristic	AAV (*n* = 125)	GPA (*n* = 50)	MPA (*n* = 25)	EGPA (*n* = 50)
Female, *n* (%)	70 (56.0)	25 (50.0)	16 (64.0)	29 (58.0)
Age at diagnosis, mean (s.d.), years	51.5 (16)	47.4 (16)	60.5 (17)	51.5 (14)
Age at enrolment, mean (s.d.), years	55.4 (15)	51.4 (16)	64.4 (15)	54.8 (13)
Ethnicity, *n* (%)				
White	116 (92.8)	46 (92.0)	24 (96.0)	46 (92.0)
Asian	5 (4.0)	4 (8.0)	0 (0)	1 (2.0)
Black	1 (0.8)	0 (0)	0 (0)	1 (2.0)
More than one ethnicity	1 (0.8)	0 (0)	1 (4.0)	0 (0)
Not recorded	2 (1.6)	0 (0)	0 (0)	2 (4.0)
Smoking history, *n* (%)				
Ex-smoker	42 (33.6)	13 (26.0)	11 (44.0)	18 (36.0)
Current smoker	2 (1.6)	2 (4.0)	0 (0)	0 (0)
Positive test for ANCA, *n* (%)	84 (67.2)	44 (88.0)	20 (80.0)	20 (40.0)
Manifestations (ever), *n* (%)				
Lung^a^	100 (80.0)	38 (76.0)	18 (72.0)	44 (88.0)
Cardiac	18 (14.4)	2 (4.0)	1 (4.2)	15 (30.0)
Neurological	53 (42.4)	15 (30.0)	4 (16.0)	34 (68.0)
Gastrointestinal	10 (8.0)	0 (0)	0 (0)	10 (20.0)
Constitutional	93 (74.4)	32 (64.0)	21 (84.0)	40 (80.0)
Musculoskeletal	81 (64.8)	29 (58.0)	12 (28.0)	40 (80.0)
Skin	44 (35.2)	16 (32.0)	4 (16)	24 (48.0)
Ophthalmological	23 (18.4)	17 (34.0)	0 (0)	6 (12.0)
ENT	52 (41.6)	42 (84.0)	4 (16)	6 (12.0)
Renal	57 (45.6)	27 (54.0)	22 (88.0)	8 (16.0)
Thrombosis	14 (11.2)	3 (6.0)	4 (16.0)	7 (14.0)
Laboratory tests				
Haemoglobin, mean (s.d.), g/dl	13.3 (2)	13.5 (2)	12.6 (2)	13.4 (2)
White blood cells, mean (s.d.), 10^9^/l	8.6 (3)	7.9 (2)	8.7 (2)	9.6 (5)
Platelets, mean (s.d.), 10^9^/l	282 (93)	277 (100)	258 (88)	296 (86)
CRP, mean (s.d.), mg/l	18.3 (18)	19.0 (19)	22.8 (14)	15.4 (18)
Vitamin D status				
25(OH)D level, mean (s.d.), ng/ml	37.6 (16)	35.9 (20)	39.2 (15)	38.6 (12)
Sufficient, *n* (%)	86 (68.8)	28 (56.0)	19 (76.0)	39 (78.0)
Insufficient, *n* (%)	26 (20.8)	15 (30.0)	4 (16.0)	7 (14.0)
Deficient, *n* (%)	13 (10.4)	7 (14.0)	2 (8.0)	4 (8.0)
Summer^b^ 25(OH)D level, mean (s.d.), ng/ml	36.0 (14)^d^	–	–	–
Winter^c^ 25(OH)D level, mean (s.d.), ng/ml	38.6 (17)^d^	–	–	–
Relapse history and disease activity				
Relapse(s) before enrolment, *n* (%)	48 (38.4)	23 (46.0)	7 (28.0)	18 (36.0)
Remission at time of enrolment, *n* (%)	79 (63.2)	30 (60.0)	18 (72.0)	31 (62.0)

a Includes asthma.

b June–October, *n* = 45.

c November–May, *n* = 80.

d *P*  = 0.409.

AAV: ANCA-associated vasculitis; EGPA: eosinophilic granulomatosis with polyangiitis; GPA: granulomatosis with polyangiitis; MPA: microscopic polyangiitis.

Relapse before enrolment occurred in 48 patients (38.4%), and 79 (63.2%) were in remission at enrolment. Characteristics of the subset of patients (*n* = 21) who relapsed after enrolment are detailed in Table 2.

**Table 2.** Clinical characteristics of 21 patients with ANCA-associated vasculitis at study enrolment and subsequent relapse visits

Characteristic	Enrolment visit (*n* = 21)
Female, *n* (%)	12 (57.1)
Age at enrolment, mean (s.d.), years	51.4 (17)
Positive test for ANCA, *n* (%)	15 (71.4)
Diagnosis, *n* (%)	
GPA	16 (76.2)
MPA	1 (4.8)
EGPA	4 (19.0)
Manifestations (ever), *n* (%)	
Lung	20 (95.2)
Cardiac	2 (9.5)
Neurological	8 (38.1)
Gastrointestinal	1 (4.8)
Constitutional	12 (57.1)
Musculoskeletal	11 (52.4)
Skin	10 (47.6)
Eyes	4 (19.0)
ENT	16 (76.2)
Renal	9 (42.9)
Thrombosis	3 (14.3)
History of relapse before enrolment, *n* (%)	12 (57.1)

		Relapse visit	*P*-Value
Enrolment duration at relapse, mean (s.d.), months		10.4 (6)	
Vitamin D status			
25(OH)D level, mean (s.d.), ng/ml	37.8 (16)	38.0 (10)^a^	0.922
Sufficient, *n* (%)	15 (71.4)	17 (81.0)	
Insufficient, *n* (%)	3 (14.3)	3 (14.3)	
Deficient, *n* (%)	3 (14.3)	1 (4.8)	
Summer 25(OH)D level, mean (s.d.), ng/ml	41.9 (15)^d^	37.1 (10)^e^	
Winter 25(OH)D level, mean (s.d.), ng/ml	35.7 (16)^d^	39.2 (8)^e^	

a Serum within 3 months of the relapse visit.

b June–October, *n* = 12.

c November–May, *n* = 9.

d *P*-value = 0.425.

e *P*-value = 0.621.

EGPA: eosinophilic granulomatosis with polyangiitis; GPA: granulomatosis with polyangiitis; MPA: microscopic polyangiitis.

### Treatments

Glucocorticoid-sparing agents ever used among patients in the study cohort included: CYC (54.4%), AZA (36.8%), MTX (28.8%), rituximab (19.2%), MMF (9.6%), CSA (0.8%) and mepolizumab (0.8%). Eight (6.4%) patients were off all treatment. Of 109 patients with data available on use of glucocorticoids, 87 patients (79.8%) were taking systemic glucocorticoids at the time of enrolment.

### Vitamin D status and its association with clinical characteristics

The mean vitamin D level at study enrolment was 37.6 (16) ng/ml, with 26 patients (20.8%) insufficient and 13 patients (10.4%) deficient in vitamin D. Sufficient levels were observed in only 28 (56%) patients with GPA, compared with 19 (76%) with MPA and 39 (78%) with EGPA, but these differences were not statistically different (*P *= 0.167). There was also no difference in vitamin D based on summer *vs* winter periods (*P *= 0.409).

Univariate analysis found the vitamin D level to be lower in patients with active disease [33.9 (2) ng/ml] compared with patients in remission [39.9 (2)ng/ml; *P* = 0.047; Supplementary Fig. S1, available at *Rheumatology Advances in Practice* online], and in males [34.0 (2) ng/ml] compared with females [40.5 (17) ng/ml; *P* = 0.027; Supplementary Table S2, available at *Rheumatology Advances in Practice* online]. Deficient vitamin D status (<20 ng/ml) was also correlated with disease activity in both univariate (*P* = 0.015) and multivariate analysis (*P* = 0.017; Supplementary Table S1, available at *Rheumatology Advances in Practice* online). The remaining variables analysed were not correlated with vitamin D level or age, diagnosis, geographical location, ANCA status, ethnicity, CRP, haemoglobin, WBC, platelets, glucocorticoid use, time from diagnosis to enrolment, kidney disease or history of relapse (Supplementary Tables S1 and S2, available at *Rheumatology Advances in Practice* online).

In the 21 patients who relapsed after study enrolment [10.4 (6) months after study enrolment], there was no significant difference between the vitamin D status at enrolment [37.8 (16) ng/ml] and at the time of the relapse visit [38.0 (10) ng/ml; *P* = 0.922; Supplementary Fig. S2, available at *Rheumatology Advances in Practice* online].

## Discussion

This study showed that one-third of the patients with AAV in this North American cohort had insufficient or deficient vitamin D status. In this cohort, male sex and disease activity were associated with lower 25(OH)D_2/__3_ levels. However, there was no correlation between relapses and vitamin D status at the individual patient level.

Few studies have been conducted on vitamin D levels in patients with vasculitis. Previous studies commonly found vitamin D deficiency to be prevalent in various vasculitides. A cohort from Istanbul (Turkey) of patients with AAV (*n* = 42) and other small-to-medium vasculitides (*n* = 15) found vitamin D deficiency/insufficiency in 74% of patients [9]. Likewise, 54 patients with AAV from South Korea had lower 25(OH)D_3_ levels when compared with 50 age- and sex-matched healthy controls (16.0 *vs* 20.4 ng/ml, *P* = 0.016) [11]. Lower 25(OH)D_3_ levels [16.9 (10) nmol/l] were also seen more often in patients with Takayasu’s arteritis than in patients with Behçet’s disease [38.8 (20) nmol/l] and healthy controls [64.6 (22) nmol/l] [8]. Finally, in a cohort of Brazilian patients with PMR/GCA, 25(OH)D_3_ deficiency occurred in 13% of patients [14]. In the present study, we found a low mean vitamin D level in 125 patients with AAV, but the rate of deficient 25(OH)D_3_ status was comparable to that in both Canadian and American-based general populations [15, 16]. This observation might be because North Americans largely depend on fortified foods and dietary supplements to meet vitamin D needs, along with population-based education on the importance of vitamin D.

In the present study, we found no significant differences in vitamin D status when comparing northern *vs* southern study sites, probably owing to limited differences in latitude with similar seasons at the locations of the study sites. As a surrogate of the duration taking vitamin D supplementation (common practice in AAV to prevent glucocorticoid-related osteoporosis), time from diagnosis to enrolment visit was analysed and showed no association with vitamin D status. Association of 25(OH)D_3_ levels with season was also not found to have a significant difference, probably owing to limited power in the present study because of small sample sizes for each subset and variation of exogenous vitamin D intake.

A numerically (but not statistically) greater proportion of patients with GPA had insufficient or deficient vitamin D status, when compared with MPA and EGPA. This might be related to disease-specific variations and/or possibly signal an interplay of vitamin D status and disease activity, and the higher incidence of disease relapse seen in GPA. No specific disease manifestations (including kidney) were associated with vitamin D status in the VCRC cohort, in contrast to Korkmaz *et al.* [9], who identified renal involvement to be associated with vitamin D deficiency/insufficiency [odds ratio 22.5 (95% CI 1.6, 128.9)]. However, male sex (univariate) and disease activity (univariate and multivariate analysis) were associated with lower levels of vitamin D. Although male sex is not a known risk factor for vitamin D deficiency, one study found it to be prevalent in older males (>80 years), particularly if they were obese, had a sedentary lifestyle or lived at higher latitudes [17]. Disease activity in AAV has previously been negatively correlated with vitamin D status when using the BVAS, CRP and WBC [11]. Likwiese, a meta-analysis including 939 participants with Behçet’s disease identified an association between vitamin D deficiency and active disease [18].

Despite this association between lower vitamin D level and disease activity at enrolment for the entire study group, there was no significant association between vitamin D status at the individual patient level between enrolment and subsequent relapses. Several reasons might account for this lack of association, including seasonal variation, routine prescription of vitamin D_3_ supplementation and adherence, small number of patients analysed at the time of disease relapse and/or the onset of flare did not always correspond to the relapse study visit (weeks to months between the flare and blood sampling). Prospective studies are needed to determine whether optimization of vitamin D status impacts disease activity and risk of relapse in AAV.

The strengths of this study include standardized data collection from a large cohort of patients with AAV, across multiple centres in the USA and Canada. Limitations of this study include the retrospective design, the lack of information on vitamin D supplementation, dietary intake of vitamin D, sun exposure or use of sun protection. The dose of immunomodulatory medications (including glucocorticoid doses) at the time of sera collection was also not recorded. Finally, as a recurrent, general and unsettled debate, 25(OH)D_3_ ranges for sufficient, insufficient and deficient values were established decades ago, are controversial and might not apply uniformly to all countries, ethnicities and ages [13].

The findings of the present study support the potential role of vitamin D in the pathophysiology of autoimmune diseases, including AAV. In the last two decades, vitamin D has been found to interplay with immunomodulation at multiple levels. Vitamin D receptors were, for example, found to be expressed in almost all immune cells, except B cells [19]. Studies suggest that vitamin D deficiency has a significant role in initiation, progression and/or severity in various autoimmune conditions [19]. Increasing latitudes, decreasing UV radiation levels, and winter months have been associated with increased incidence and relapse risk in AAV [20]. Vitamin D status might be a marker of more active disease in this population, as demonstrated with these results. Vitamin D status should be obtained routinely in patients with AAV, whether on glucocorticoids or not, and if they are deficient or insufficient, they should be monitored for adherence to vitamin D supplementation. Larger studies could help to determine whether ensuring adequate vitamin D supplementation and/or a sufficient vitamin D status could alter the disease course, improve disease activity and/or reduce relapse risk in AAV.

## Supplementary Material

rkad021_Supplementary_Data

## Data Availability

The data underlying this article are available in the article and in its online supplementary material. Additional data is available upon request to the study investigator.
