# Supplementary material for: Vitamin D status in ANCA-associated vasculitis
Source: Rheumatol Adv Pract. 2023 Feb 10;7(1):rkad021. doi: 10.1093/rap/rkad021 (PMC9977244; doi:10.1093/rap/rkad021)
Supplement: rkad021_Supplementary_Data [file rkad021_supplementary_data.docx]

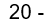

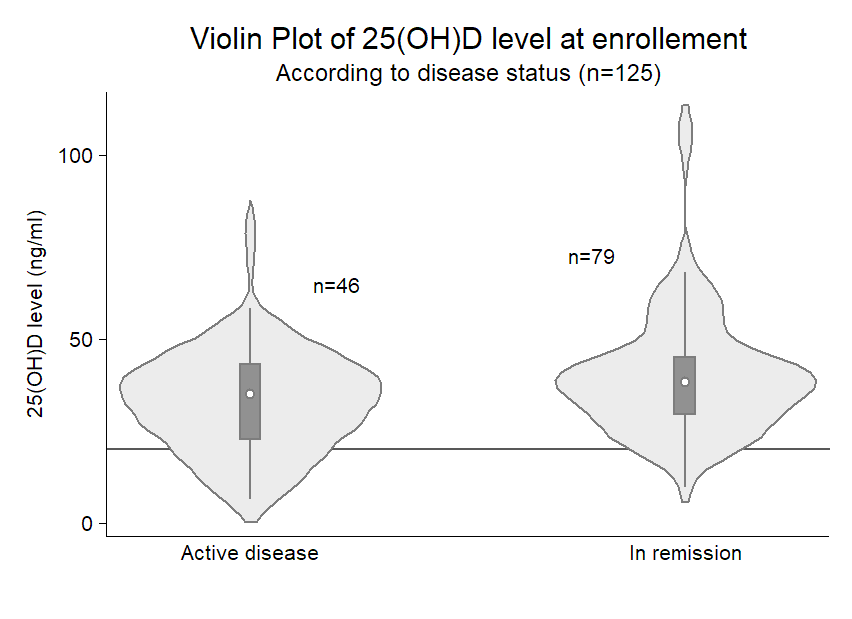


*p=0.047*

**Supplementary Figure S1.** 25(OH)D levels for 125 patients with ANCA-associated vasculitis at enrollment, according to disease status. The diamond-shape areas represent the distribution (number of patients) at each level of vitamin D. The box plots in the center of the diamond-shaped areas represent the medians (circles), and interquartiles (upper and lower box lines). Deficiency is defined as <20ng/ml (horizontal bar).

*p=0.922*

**Supplementary Figure S2.** 25(OH)D levels at enrollment and relapse visit in the 21 patients with ANCA-associated vasculitis who relapsed following study enrollment.

Supplementary Table S1. Variables associated with deficient vitamin D status from logistic regression models in patients with ANCA-associated vasculitis.

| **Variables** | **Deficient vitamin D status**  **(<20 ng/mL), n (%)** | **Univariate Analysis**  P-value | **OR**  **(95% CI)** | **Multivariate Analysis**  P-value |
| --- | --- | --- | --- | --- |
| Age | - | 0.288 |  |  |
| Race  White  Asian  Black  >1 race | 11 (9.5)  1 (100)  0 (0)  1 (100) | ­  0.096 | -^a^ | -^a^ |
| Sex  Male  Female | 8 (14.5)  5 (7.1) | 0.240 |  |  |
| Diagnosis  GPA  MPA  EGPA | 7 (14.0)  2 (8.0)  4 (8.0) | 0.691 |  |  |
| Geographic Location  North  South | 10 (12.0)  3 (7.1) | 0.541 |  |  |
| ANCA Status  Positive  Negative | 11 (13.1)  2 (4.9) | 0.218 | 1.01  (-0.58 to 2.61) | 0.213 |
| C-reactive protein | - | 0.067 | 0.01  (-0.01 to 0.03) | 0.268 |
| Hemoglobin | - | 0.413 |  |  |
| White blood cells | - | 0.765 |  |  |
| Platelets | - | 0.677 |  |  |
| Glucocorticoid use  Yes  No | 10 (11.5)  2 (9.1) | 1.00 |  |  |
| Kidney  Yes  No | 5 (8.8)  7 (10.4) | 1.00 |  |  |
| Time to diagnosis | - | 0.301 |  |  |
| Remission  Yes  No | 4 (5.1)  9 (19.6) | 0.015 | -1.52  (-2.76 to -0.28) | 0.017 |
| Relapse history  Yes  No | 4 (8.3)  6 (8.1) | 1.00 |  |  |

*ANCA: Antineutrophil cytoplasm antibody. GPA: granulomatosis with polyangiitis; MPA: microscopic polyangiitis; EGPA: eosinophilic granulomatosis with polyangiitis.*

*^a^Race was not included due to skewed data set from lack of minorities.*

Supplementary Table S2. Variables associated with vitamin D level from logistic regression models in patients with ANCA-associated vasculitis.

| **Variables** | **Vitamin D level, mean±SD (ng/mL)** | **Univariate Analysis**  P-value | |
| --- | --- | --- | --- |
| Age | - | | 0.101 |
| Race  White  Asian  Black  >1 race | 37.5±16  40.3±16  68.1±0  10.0±0 | | ­  0.163 |
| Sex  Male  Female | 34.0±15  40.5±17 | | 0.027 |
| Diagnosis  GPA  MPA  EGPA | 35.9±20  39.2±15  38.6±12 | | 0.617 |
| Geographic Location  North  South | 36.5±16  39.9±17 | | 0.275 |
| ANCA Status  Positive  Negative | 36.5±17  39.9±14 | | 0.276 |
| C-reactive protein | - | | 0.152 |
| Hemoglobin | - | | 0.480 |
| White blood cells | - | | 0.814 |
| Platelets | - | | 0.344 |
| Glucocorticoid use  Yes  No | 35.9±15  40.3±19 | | 0.241 |
| Kidney  Yes  No | 36.2±17  39.4±16 | | 0.272 |
| Time from diagnosis to enrollment | - | | 0.148 |
| Remission  Yes  No | 39.9±17  33.9±15 | | 0.047 |
| Relapse history  Yes  No | 39.6±18  37.3±15 | | 0.452 |

*ANCA: Antineutrophil cytoplasm antibody; GPA: granulomatosis with polyangiitis; MPA: microscopic polyangiitis; EGPA: eosinophilic granulomatosis with polyangiitis*
